# Lecture upon Lycopodium

**Published:** 1888-09

**Authors:** J. T. Kent

**Affiliations:** Philadelphia


### LECTURE UPON LYCOPODIUM.

PROF. J. T. KENT, A. M., M. D., PHILADELPHIA.

Through the genius of Hahnemann, the dynamic and sick-making powers of Lycopodium—heretofore believed to be an inert drug—have been brought out, and we find it to be a long, deeply-acting remedy, having a broad sphere of action. It is an important anti-psoric, being one of the polychrests. One dose of Lycopodium given in a high potency will ward off the symptoms of a chronic miasm for a long time, and many times cure a chronic disease; if after a long time symptoms of disturbance of this chronic miasm should arise, another dose of different and higher potency will cause the subsidence of such conditions and symptoms for another long period.

Lycopodium acts powerfully upon the tissues and fluids of the body, causing diseased conditions resembling psora; especially such psoric effects as seem to have been handed down through generations, causing low forms of disease; active and progressive conditions of chronic disease; corrosive forms of disease, such as we find in corroding ulcers of the stomach; eruptions like epithelioma; infiltrations beneath ulcers; manifestations of warty growths; of *all* kinds of sores and eruptions; of low, anemic conditions. In this you see how deeply Lycopodium attacks the vitality. Like Carb. veg., Chin., Ars., Larant., it has all the signs of threatened dissolution.

There is great regularity of manifestation of the *aggravations* of this medicine. Most of its complaints, its pains, its febrile conditions, its coldness are *agg.* in the afternoon and evening, from four to eight o'clock; this is a strong characteristic and belongs to the *red string.* There is also *agg.* from cold; the complaints and irritations of the mucous membranes, the skin, and the general conditions are *agg.* from cold. Cold atmospheric changes; exposure to cold air. Cold drinks *agg.* The latter seems to make worse both the throat and stomach symptoms. Warmth is in general comforting in most of the Lycopodium complaints; yet there is some *agg.* from becoming heated in the open air. Hot drinks relieve the sore throat and the irritation of the stomach.

Lycopodium produces the hippocratic countenance; the pallid, pinched face, with sweat, blueness, deathly sickness, rings about the eyes, sinking in about the nose, fan-like motion of the alæ nasi, abdominal breathing, heaving of the chest, feeble heat, coldness of the extremities; all symptoms of the gravest character.

Lycopodium has the febrile state; it has the chill; it has the sweat; it has all the manifestations of inflammation, and passes from the early inflammatory states to that of abscesses, indurations, ulcerations, destruction of tissues, and molecular death. It has all the common features of sepsis, zymosis, and diphtheria. It has the septic signs, the prostration, the profuse sweating, the frequent small pulse, the sinking and the suffocation. It goes deep into life and brings about changes that are remarkable, particularly in the zymotic state, the zymotic process. The exudations on the teeth, the tympanitic abdomen, the frequent pulse, the weak heart, the general irritable state, the typhoid conditions so often quoted, with diarrhœa and ulceration. As I have before mentioned, it has the diphtheritic process; the exudations of diphtheritic character upon the throat, or wherever it may elect to appear—in the rectum, in the vagina, in the nose, or in the larynx.

Most of its complaints travel from right to left; its rheumatisms belong mostly to the right side, traveling to the left; its exudations form on the right side and then on the left; its paralytic conditions belong to the right side with a tendency to travel to the left; the opposite, therefore, of Lachesis—which is from left to right; the right ovary, from the right ovary to the left is Lyc., and pain in the left ovary, traveling to the right, is Lachesis.

Suppurative conditions form marked indications for this remedy. It has been very valuable in suppuration of the lungs; in pus cavities resulting from septicæmias and in abscesses. It is also allied to the gangrenous states; threatened gangrene; gangrenous inflammation; patulous appearances that are bluish and gangrenous, or that threaten to become such, associated with septic signs, and the general typhoid state.

Another peculiarity of Lycopodium is the *offensiveness* of its discharges; the sweat, the stool, and even the breath are offensive.

Again, a prominent feature is its glandular enlargements, especially enlargement of the glands about the neck; enlarged tonsils; enlarged lymphatics; enlargements with indurations and with suppurations.

Flatulency is a marked characteristic of Lycopodium; much flatus, which may pass either way, with marked distention of the abdomen; abdomen distended even after a slight meal; belching does *not* relieve; with China, Lycop., and Nux, warm drinks *amel.* in the abdominal complaints; with Lyc. warm drinks and the nausea caused by cold water. Chin. has flatulency from tea drinking. Flatulency is a disturbing element in most chronic cases demanding Lyc. often. Associated with great mental weakness, confusion and distraction of mind.

Lycopodium has a wonderful range of urinary symptoms; pains, aches, and annoyance in connection with the bladder and kidneys. The urine is turbid and bad smelling, and if allowed to stand for a while, red sand appears as a deposit. The child cries before passing water.

There are muco-purulent discharges from mucous membranes of the bladder, of the urethra, of any or all the mucous membranes of the body. Its character is thick, yellowish, may be greenish. We find catarrhal conditions of the chest with this thick, yellow, purulent or muco-purulent discharge, to be very common to Lyc. The sputa is salt to the taste. It has also a dry catarrhal condition, dry and burning; the mucous membrane is in a state of irritation; this condition may exist in nose or chest, or it may exist in the throat or the pharynx. We may also find a dry, teasing cough. Here is a key-note, *dry, teasing cough in emaciated boys.*

Lycopodium competes with Sepia in spots upon the skin; yellow spots, brown spots, or brownish-yellow spots; they are sometimes quite dark brown.

Another grand feature of this remedy is its paralytic condition—its paralytic weakness. We find paralysis of the limbs; paralysis of the brain; great weakness of brain power; the brain entirely giving out, becoming exhausted, preceded by distraction; it is one of our greatest remedies in brain-fag; weakness of the mind. We also find paralysis of the limbs, and with all paralysis of the sexual organs; impotency. The sexual organs become debilitated and cold. A general index for the remedy in these conditions would be—the limbs and extremities become cold, with feeble circulation; anæmia of the spinal cord.

This remedy has many features like Rhus, it has great restlessness like Rhus, it is relieved by motion like Rhus. Rhus corresponds to a state that has arisen suddenly; to such as arise in the early part of the case, sometimes indeed to a very old case. Lyc. would not be as likely to correspond to a case in the early stages, for it is an inert substance, and a slow-acting drug, bringing about complaints after a long time; after long and insidious working, bringing about this disintegration or breaking down of tissue; tissue changes; hence, long after Rhus has become inactive or ineffectual, Lyc. is thought of—but only because of its own symptoms. These are about the only features similar to Rhus; the paralytic condition; the rheumatic affections, and the great restlessness; made better from motion.

Lyc. has dropsical swellings also as a characteristic, particularly a swelling of the lower extremities. There is pain of the legs, or of the knees and feet, both; hepatic dropsy; hence a filling up of the peritoneum with fluid; engorgement of the liver, finally bringing about atrophy, and a nodular condition of the liver.

Lyc. produces varicose veins here and there, especially upon the lower extremities. It not only has atrophy of the liver, but it has shrinking of other parts, and it has marked emaciation, with a dry cough. It has a characteristic emaciation from above downward, wherein it competes with Nat-mur. The emaciation about the neck and shoulders, progressing downward, may be the first emaciation you will notice about a Lycopodium patient. There is one remedy of which we have spoken, that is peculiar in its emaciation about the diseased part: that is Pulsatilla. The diseased limb withers; with this he has inclination for the open air. The headache; the chest symptoms when present, give him the desire for cold air, for fresh air, and he is much disturbed over warmth even though chilly. An inclination for the open air, yet he is generally cold and chilly. This helps you to understand Lyc.; the headache is generally better from cold, while the general bodily states are better from warmth. Headache *agg.* by warmth and by getting warm while walking; from mental exertion; *amel.* by the open air; in cold air; and from uncovering the head. This is the exception regarding the cold; most other complaints are better from warmth.

There is a guiding symptom in relation to the back; the *burning* as of hot coals between the scapulæ. Compare with Phosphorus. Glonoin. has burning like hot coals the whole length of the spinal cord. Pains and aches across the spleen and lumbar region are quite characteristic of Lyc. Many remedies have these pains and aches across the small of the back, but very few of them have these pains and aches across the small of the back, relieved by passing the urine. Many of the symptoms of Lyc. come on *before*, and are relieved by urinating.

This much belongs to the strong characteristics: the whole symptom picture; the pathogenesis—in fact, the complete red string of the remedy. With that red string you will be able to cure many complaints. Having a good portion of the general state of the remedy you must also know that the special symptoms are present, but you *may have all the special symptoms without these grand, general features, and not succeed in curing your patient.* The mental symptoms of Lycopodium evidence a marked weakness and relaxation. There is great weakness and relaxation of every part of the body, hence the patient is disturbed in mind. In brain-fag, a tired mind, this is a great remedy. The nervous exhaustion, with enfeebled mind, takes various shapes. In some cases the patient is afraid to be left alone, even afraid of his own shadow; dread of men. This dread of men, or dread of the opposite sex, is often associated with impotency and aversion to sexual intercourse. It is common for the patient to say she is afraid to be left alone; she may be afraid something is going to happen; fears of various kinds come upon her; there is forgetfulness, anguish, and excitement when alone. (Ars., Bism. have great fears and forgetfulness when alone. The Phos. patient is afraid something is going to happen when alone in a room, especially at night. The Arg-nit. patient hardly dare remain alone lest he harm himself, fears and anxiety will compel him to move about. He also fears to go upon a high bridge or lofty place lest he throw himself down. This is a marked symptom of Arg-nit. Anac. has the same.)

The Lycopodium patient uses the wrong word from mere weakness; he knows that he does so and tries to correct himself, often repeating the blunder (compare with Dulc.); feeble mind; absent mind; great melancholy; sadness, with confusion of mind; religious melancholy; dwelling upon religious topics; thinks he has sinned away his day of grace. Of course, these symptoms are absent in those people not of a religious turn of mind. You would hardly expect to find them in an infidel. The Lyc. patient is tearful, as well as sad—is inclined to weep a great deal, even as much as the Puls. patient; is tired of life; wants to die; has distaste and disgust for life; her surroundings are disgusting to her and she is always tired and worn out. At times she is indifferent to her family and will not answer questions; does not wish to be annoyed; wishes to be alone and quiet, and still there comes the fear when she is alone, so she is not contented anywhere. This partakes of the general restlessness running through the drug, sometimes benefited by moving about. (Restlessness, like Rhus and Puls., but the latter is *amel.* by slow motion. Any kind of motion relieves Rhus.)

The patient is willful in Lyc. and will not obey, headstrong; wants her own way, and withal very deceitful.

The irritability, resulting in fear, is sometimes so marked that a strong impression is made upon the body. There exists in the body an apprehensiveness, a modified fear, especially when alone, which creates an increased susceptibility to a natural cause for fear, so that if the natural cause arises it produces a profound impression upon the body, *i. e.*, unconsciousness; unconsciousness, with spasms, after which follow disease of organs; affections of the heart; affections of the bowels, particularly engorgement of the liver. Both acute and chonic disease have localized themselves upon the liver because of this fear. Mental states resultant from fear frequently resemble those of Igt., Hyos., and Act-rac., but we have diseases of organs, especially of the liver, brought about by fear. A frontal congestion, with all its sequences, may come about by being frightened, by being greatly disturbed, and by suddenly coming upon something frightful or horrible.

There is more or less of dizziness in Lyc., and this remedy can furnish you with most any kind of head pain. The peculiarity of the head pains is the *time* and the *kind* of *agg.* The headaches are worse from four to eight in the evening and by eating, for there are many stomach symptoms in relation with Lyc.—*amel.* in the open air, in a cool place, or by uncovering the head (Ars. is similar in its general *amel.* from warmth, with head symptoms alleviated by cold) is *agg.* by warmth of bed, from becoming heated during a walk, from mental exertion, from heat in general.

There is great loss of hair; hair falling in great quantities. This also may occur after fright, after typhoid, or in syphilis if the syphilitic case has been running two or three months he then begins to loose his hair. Low forms of zymotic disease will bring out the hair, erysipelas of the scalp (in this loss of hair you will need to know Ph-ac., Lyc., Graph., Phos., Sulph., and many others, twenty or thirty of them; even Sep. relates to loss of hair.)

Eruption, beginning upon the back of the head, *crust thick* and *easily bleeding, oozing* of *fetid moisture, agg.* by *scratching* and *warmth.*

The eye symptoms are also important. There are many diseases of the eye in which you will need to know the use of *Lyc.*, peculiar perhaps are those with crusty formations upon the lid edges, having thick, yellow, muco-purulent discharge, in which the general bodily weakness, the mental symptoms, or may-hap some active symptom like red sand in the urine will be guiding, or again, the least mouthful of food causes him to feel that he has taken a full meal. Of course, if you are not looking for symptoms or used to looking for symptoms you will not find them, as many times the patient might say, "I feel well in every other way," but then you would be obliged to resort to Boracic acid solutions, Sulph. Zinc., or some other slop, and where then is your Homœopathy? Lyc. has many diseases of the eye, pustules, styes, mucus in the eye, inflammation, conjunctiva looks like raw flesh (very like Apis), copious discharges of pus, accumulations of thick yellow or yellowish-green mucus, and quite apt to be profuse.

There is a characteristic discharge from the ears with much roaring, otorrhœa, purulent, ichorous—frequently met with after scarlet fever with impaired hearing—sensation as if hot blood rushed into the ears. It is a wonderful remedy in these characteristic discharges of yellow or yellowish-green mucus from any portion of the body. Many children have the nose stuffed with thick, yellowish-green matter, cannot breathe through the nose night or day. Always stuffed up. S. L. G. L.

[CONTINUED.]

