# Errata of First Lecture of Lycopodium

**Published:** 1888-11

**Authors:** 


ERRATA OF FIRST LECTURE OF LYCOPODIUM.

Page 462, line eleven, read *Tarent.*
Page 463, line eight, for *heat* read *heart.*
Page 468, line twelve, should read *Sulphate of Zinc.*

